# Malignant retroperitoneal schwannoma in a young adult: rapid recurrence, metastasis, and treatment reflections—a case report

**DOI:** 10.3389/fonc.2025.1588573

**Published:** 2025-05-30

**Authors:** Zeming Zhao, Shaoqing Fan, Donghao Yu, Guiying Wang

**Affiliations:** ^1^ Department of General Surgery, The Second Hospital of Hebei Medical University, Shijiazhuang, Hebei, China; ^2^ Department of General Surgery, The Fourth Hospital of Hebei Medical University, Shijiazhuang, Hebei, China; ^3^ Department of Ophthalmology, The Second Hospital of Hebei Medical University, Shijiazhuang, Hebei, China

**Keywords:** retroperitoneal schwannoma, malignant schwannoma, diagnosis, treatment, prognosis

## Abstract

Retroperitoneal schwannoma, a rare mesenchymal-derived tumor originating from the retroperitoneum. Malignant retroperitoneal schwannoma carries a dismal prognosis. We present a case of a 19-year-old male who presented with left abdominal pain. Imaging examination revealed a large retroperitoneal mass (16×12×9 cm) in the left upper quadrant. Pathological examination following surgical resection confirmed the diagnosis of malignant schwannoma, with a notably high Ki-67 proliferation index of 50%. Despite radical resection, the tumor recurred with metastases to the ilium and sacrum within two years postoperatively. The patient ultimately discontinued treatment due to disease progression. This case underscores the aggressive nature of retroperitoneal malignant schwannoma, characterized by rapid local recurrence, distant metastasis, and resistance to surgical cure. These findings emphasize the urgent need for effective postoperative adjuvant therapies to improve outcomes in this highly malignant entity.

## Background

Schwannoma, a mesenchymal-origin neoplasm, arises in any neural tissue containing Schwann cells, predominantly localized to anatomical regions with dense concentrations of both central and peripheral nervous system elements, particularly the craniofacial area. Schwannomas arising in the retroperitoneum represent a rare clinical entity, constituting merely 3% of all schwannoma cases (1), with the majority exhibiting benign biological behavior. Malignant retroperitoneal schwannomas are exceptionally uncommon, accounting for 1%-2% of total schwannoma presentations (2). Early-stage retroperitoneal schwannomas frequently manifest as asymptomatic lesions (2).

Anatomically, the retroperitoneal space extends cephalad from the diaphragmatic plane to caudally merge with the pelvic compartment. This potential space maintains structural continuity through defined anatomical conduits with both the peritoneal cavity and extraperitoneal pelvic regions. The posterior boundary is demarcated by the quadratus lumborum muscle, while maintaining continuity with the retroperitoneal adipose compartment. Consequently, schwannomas in this region demonstrate potential multidirectional expansion: posteriorly toward the posterior mediastinum, laterally along the abdominal wall musculature, and inferiorly into the extraperitoneal pelvis. Such expansive growth patterns typically result in significantly larger tumor volumes compared to schwannomas occurring in other anatomical regions (3).

Advanced disease progression often leads to mass effect-induced compression of adjacent viscera, precipitating symptom complexes secondary to mechanical displacement (4). Diagnostic challenges stem from the tumor’s nonspecific morphological features on non-invasive imaging, rendering it difficult to differentiate from colonic malignancies and mesenchymal tumors. Preoperative determination of malignancy remains elusive (5), potentially compromising therapeutic decision-making and prognostic evaluation. Notably, malignant variants demonstrate a 50%-60% postoperative recurrence rate despite initial surgical intervention achieving symptomatic relief (6).

## Case presentation

**Figure 1:**
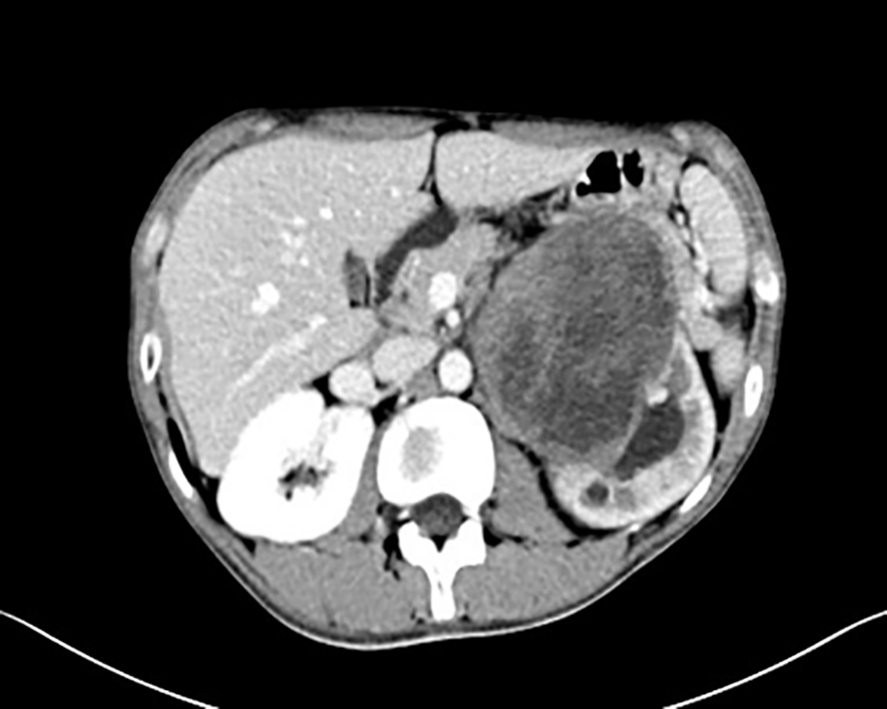
CT imaging characteristics. Intra-abdominal mass in the left upper quadrant.

A 19-year-old male presented with a 1-year history of left flank pain exacerbated over the past week. No significant constitutional symptoms were reported. The patient denied family history of hereditary disorders or neoplastic syndromes. Physical examination revealed a palpable mass (7×8 cm) in the left upper quadrant demonstrating limited mobility with well-defined margins. CT(Computed Tomography) identified an intra-abdominal mass in the left upper quadrant, radiologically suspicious for stromal tumor (**Figure 1**). Percutaneous core needle biopsy histopathology demonstrated proliferating spindle-shaped cells with fascicular arrangement. Serum tumor marker analysis revealed markedly elevated CA19-9 (>1000 U/mL; normal <30 U/mL) and marginally increased CEA (7.3 ng/mL; normal <5 ng/mL).

## Treatment

Following comprehensive preoperative evaluation excluding surgical contraindications, the patient underwent en bloc tumor resection under general anesthesia in December 2017. Intraoperative exploration identified a massive tumor (25×20×15 cm) in the left mid-lower abdomen with close anatomical relationships to the left hemicolon and the left Gerota’s fascia. We completely resected Gerota’s fascia during the operation, but complete separation from the colonic mesentery proved unfeasible. After multidisciplinary consultation, left hemicolectomy with end-to-end colorectal anastomosis was performed (operative duration: 224 minutes; EBL: 300 mL), requiring transfusion of 4 units SRBC (Suspended Red Blood Cells) and 425 mL FP (Frozen Plasma).Pathological examination revealed a 16×12×9 cm mass with firm, variegated cut surface. Immunohistochemical profile: CD34(+), CD117(-), DOG-1(-), Desmin(-), SMA(-/+), Calponin(-/+), S100(+), Ki-67 50%, MDM2(-), EMA(-). Final diagnosis favored malignant mesenchymal neoplasm with schwannomatous differentiation. All sampled lymph nodes (celiac:1, mesenteric:1, mesocolic:2) demonstrated reactive changes without malignancy. The patient recovered uneventfully and was advised to undergo periodic reexaminations and pursue aggressive treatment following the operation.

**Figure 2:**
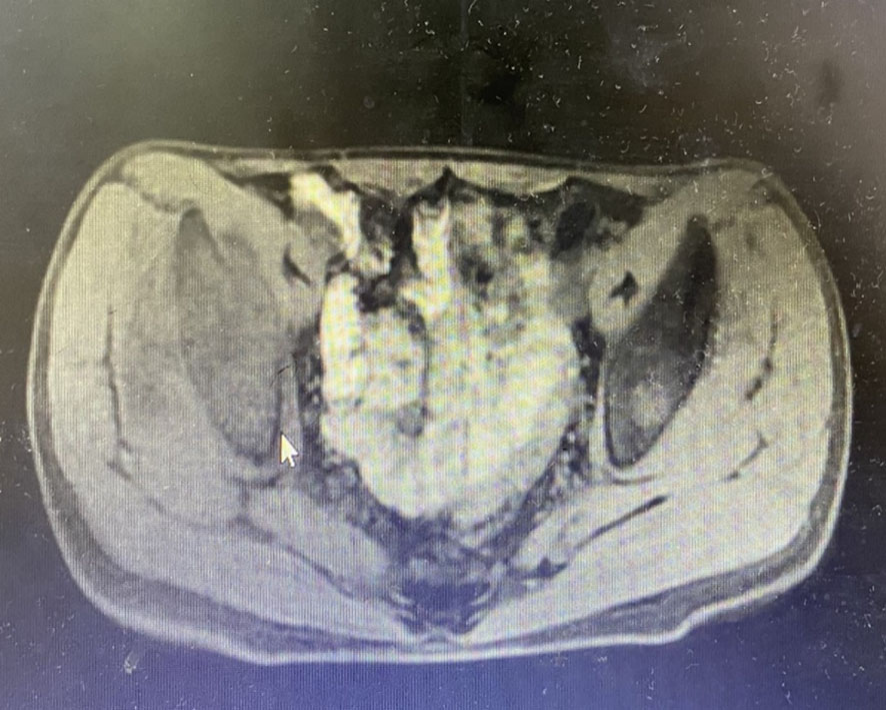
CT imaging characteristics. A 9.1×7.8 cm heterogeneously enhancing retroperitoneal mass adjacent to the left kidney.

Nonadherence to follow-up ensued until February 2019 when abdominal CT revealed a 9.1×7.8 cm heterogeneously enhancing retroperitoneal mass adjacent to the left kidney (**Figure 2**). The patient underwent left retroperitoneal mass excision with nephrectomy at an external institution. Subsequent April 2019 surveillance CT confirmed post-nephrectomy status without residual disease.

**Figure 3:**
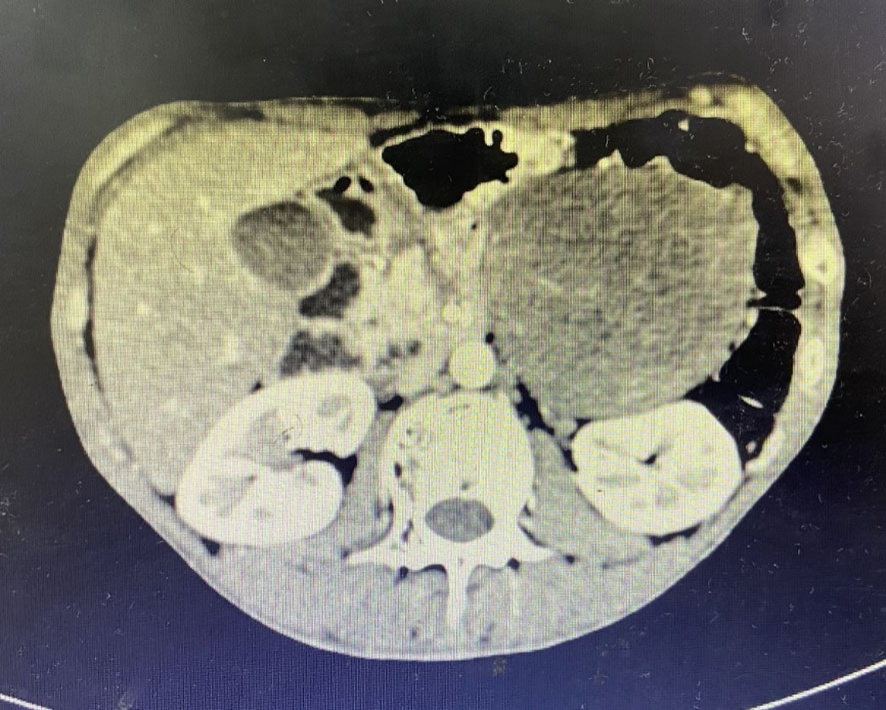
MRI imaging characteristics. Right iliac bone destruction with a 76×58×67 mm mass.

**Figure 4:**
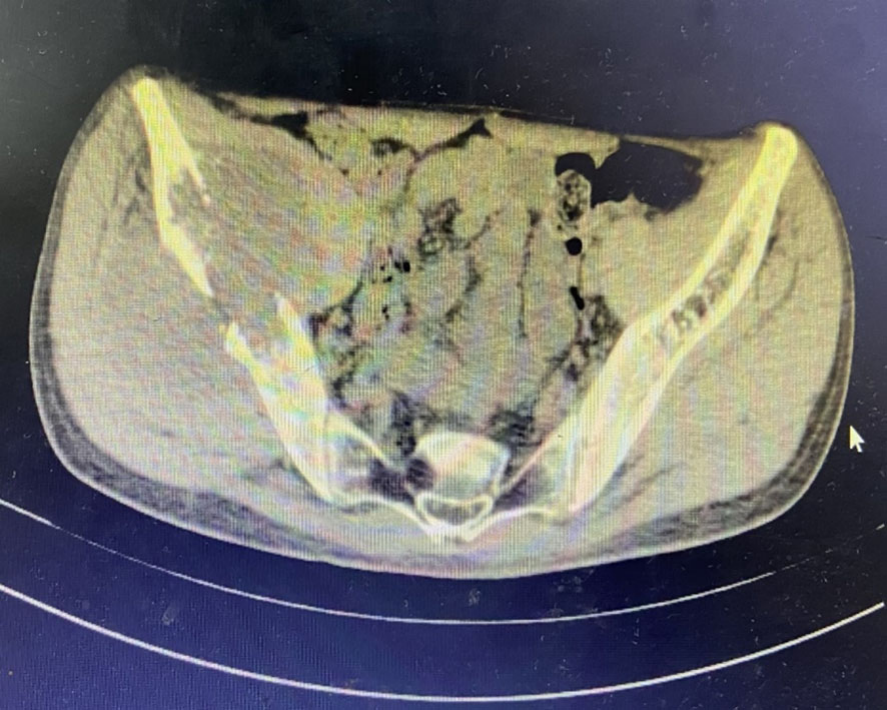
MRI imaging characteristics. Sacral and left iliac signal abnormalities.

In June 2019, the patient presented with a 3-month history of right gluteal mass and ipsilateral lower extremity weakness (muscle strength 4+/5). Pelvic MRI(Magnetic Resonance Imaging) demonstrated right iliac bone destruction with a 76×58×67 mm T1-hypointense/T2-hyperintense mass, sacral and left iliac signal abnormalities suggesting osseous metastases, and a gluteal subcutaneous cystic nodule (**Figures 3**, **4**). Upon the detection of bone metastases in this patient, a multidisciplinary team (MDT) comprising specialists from general surgery, orthopedics, interventional radiology, medical oncology, intensive care unit (ICU), radiotherapy, and pain rehabilitation was promptly assembled. The MDT focused on deliberating non-surgical treatment modalities, including chemotherapy, radiotherapy, immunotherapy, and also explored the feasibility of interventional procedures to ablate tumor tissues. Despite presenting a comprehensive array of subsequent treatment options, the patient and their family declined to proceed with further interventions, citing personal considerations.

## Discussion

Retroperitoneal schwannoma, a rare neurogenic neoplasm originating from Schwann cells, demonstrates low clinical incidence with predominant benign biological behavior. Epidemiological studies reveal a significant female predilection (7). Anatomical distribution analysis indicates gastric schwannomas demonstrate fourfold higher incidence compared to intestinal counterparts, though malignant variants exhibit predilection for abdominal cavity and small intestine rather than gastric sites (2). Benign lesions typically follow an indolent clinical course due to slow growth kinetics, whereas malignant counterparts manifest rapid tumor enlargement, invasive growth patterns, and tissue destruction within short-term progression. Clinical presentations correlate with anatomical localization, exemplified by this case’s characteristic 1-year history of persistent flank pain. Radiologically, CT imaging reveals heterogeneous density with irregular enhancement patterns, pathologically corresponding to intratumoral necrosis, cystic degeneration, and hemorrhagic changes in malignant variants. Although MRI effectively delineates peritumoral soft tissue infiltration, its diagnostic specificity for malignancy remains limited. Although some experts have proposed the use of preoperative fine-needle aspiration biopsy (FNAB) for diagnosing the nature of tumors, studies on retroperitoneal schwannomas have shown that 0.4% to 2.0% of patients who underwent the biopsy experienced needle tract seeding metastasis (8, 9). This incidence is likely underestimated due to factors such as a short follow-up period, patient loss to follow-up, and the inherent difficulty in accurately assessing intra-abdominal seeding. Therefore, the routine application of preoperative needle biopsy remains highly controversial. For patients for whom surgical resection is the preferred treatment option, concerns regarding the potential oncological risk of seeding outweigh the diagnostic benefits of this examination (10).

Definitive diagnosis requires histopathological examination supplemented by immunohistochemical profiling, with hallmark features including coexistence of Antoni A areas (hypercellular palisading arrangements) and Antoni B regions (myxoid hypocellular stroma), coupled with strong diffuse S-100 protein immunoreactivity (11).Malignant differentiation necessitates combined morphological and molecular evaluation: benign lesions exhibit strong uniform S-100 expression, while malignant counterparts demonstrate attenuated immunopositivity accompanied by elevated Ki-67 index (>10%), aberrant p53 expression, and HE-staining features including marked cellular atypia, increased mitotic activity (≥5/10 HPF), and infiltrative growth patterns. Notably, intraoperative frozen section analysis demonstrates limited reliability in malignancy determination, and the diagnostic utility of preoperative biopsy remains controversial due to sampling limitations (11, 12).

Radical surgical resection with tumor-free margins constitutes the primary therapeutic intervention, though malignant variants demonstrate high propensity for local recurrence (40-60%) and distant metastasis (20-40%) (13). Prognostic determinants include tumor diameter (>5 cm), margin status (R0 vs R1/R2), and histological grade, with FNCLCC grading system validated as an independent prognostic factor (14). Adjuvant radiotherapy may reduce local recurrence rates (15-25% risk reduction), though no survival benefit has been established through randomized trials (15). Initially, adjuvant radiotherapy and other therapeutic options were contemplated for this patient. Nevertheless, contemporary clinical guidelines explicitly discourage the routine use of local radiotherapy for patients with completely resected tumors (16). A comprehensive assessment of the patient’s age, Karnofsky Performance Status (KPS), comorbidities, and physiological reserve, coupled with a risk-benefit analysis, indicated that radiotherapy would confer limited clinical benefit while potentially exposing the patient to unnecessary treatment-related toxicities. Consequently, this therapeutic modality was judiciously excluded from the treatment plan. In conclusion, this disease paradigm underscores the necessity for multimodal assessment protocols, particularly emphasizing long-term surveillance (minimum 10-year follow-up) for recurrence monitoring and outcome optimization.

## Conclusion

Malignant retroperitoneal schwannoma is rare and highly invasive. Radical surgery is the only effective treatment, but the risks of recurrence and metastasis are high. This case indicates that early identification of malignant features, standardized postoperative management, and exploration of novel adjuvant therapies are the key directions for improving prognosis. Moreover, it is recommended that the multidisciplinary team (MDT) collaboration model be adopted in future diagnosis and treatment processes to optimize the surgical plan.

## Data Availability

The original contributions presented in the study are included in the article/supplementary material. Further inquiries can be directed to the corresponding author.
